# Editorial: Micro- and macronutrient malnutrition in cardiovascular disease

**DOI:** 10.3389/fcvm.2023.1191982

**Published:** 2023-05-04

**Authors:** J. P. Hobkirk, G. Aubert, N. Bomer

**Affiliations:** ^1^ Faculty of Health Science, University of Hull, Hull, United Kingdom; ^2^ Division of Cardiology, Stritch School of Medicine, Loyola University Chicago, Chicago, IL, United States; ^3^ CSL Vifor, Glattbrugg, Switzerland; ^4^ Department of Cardiology, University Medical Center Groningen, University of Groningen, Groningen, Netherlands

**Keywords:** obesity, malnutrition, micronutrients, undernourishment, ultra-processed food

**Editorial on the Research Topic** Micro- and macronutrient malnutrition in cardiovascular disease

According to the European Society of Clinical Nutrition and Metabolism (ESPEN), malnutrition can be defined as “a state resulting from lack of intake or uptake of nutrition that leads to altered body composition (decreased fat free mass) and body cell mass leading to diminished physical and mental function and impaired clinical outcome from disease”. Malnutrition can result from starvation, disease or advanced ageing (e.g., >80 years), alone or in combination (1). The World Health Organization (WHO) classifies malnutrition as one of the biggest threats to public health. Identifying patients at risk or with malnutrition can be undertaken by numerous screening tools, for example, the Malnutrition Universal Screening Tool (MUST) which utilises BMI, acute weight loss and loss of appetite. Blood biomarker panels have also evolved comprising of clinically adoptable measures (e.g., albumin, t-lymphocytes, ferritin, cholesterol).

In patients with acute coronary syndrome and heart failure, there is a high prevalence of malnutrition, assessed post-hoc using blood biomarker panels (2, 3). In these studies, there was no direct comparisons with other screening tools (MUST and other physical clinical assessments, e.g., sarcopenic obesity. Understanding the limits of agreements between screening tools and blood biomarker panels are urgently required, particularly where a large number of patients are normal or overweight and are typically not characterised as at risk of malnutrition *via* MUST classification. For example, Roubin et al. (2) reported the prevalence of moderate or severe malnutrition in patients with acute coronary syndrome based on three scores (CONUT, NRI, PNI). The prevalence was calculated to be 11.3%, 39.5% and 9.0% respectively. The higher observed prevalence of malnutrition was obtained with the NRI tool, which includes excess weight in its calculation (i.e., actual—ideal weight). This does raise an important question; does obesity really matter when considering nutritional risk?

Nutrient deficiencies associated with obesity may be partly due to overconsumption of foods that have a high caloric value but have a low nutrient density. Cross-sectional data from the UK National Diet and Nutrition Survey (2008–2014) in 9,364 individuals indicated that ultra-processed foods accounted for 57% of total energy intake. In that study a 10% increase in the consumption of ultra-processed foods linked with a 18% rise in the prevalence of obesity in both sexes (4). A recent systematic review/meta-analysis discussing trends of ultra-processed food consumption has underlined the importance of undernourishment in chronic noncommunicable diseases (5). Traditionally, global health has focused on two distinct issues in nutrition; overnutrition, which includes being overweight or obese; or undernutrition, which includes underweight, frailty or having nutrient deficiencies. However, both conditions can be seen in the same individual, a “double burden of malnutrition” (DBM) by increased consumption of ultra-processed food, which is considered addictive based on established scientific criteria (6), is nutrient density poor and is displacing traditional dietary habits and practices.

It is important to recognise nutritional inadequacies/deficiencies in patients regardless of BMI classification. In the US NHANES study, the population prevalence not achieving estimated average requirement (EAR) in vitamins A, C, D, E, calcium and magnesium were significantly higher moving up the BMI classifications, but still worryingly high even in the normal weight group (7). Methodologically, there are precision issues with the subjective recall of dietary intake. Additionally, physiological concentrations are mediated *via* the absorption, distribution and metabolism of the micronutrient.

There has been a proposal to harmonise nutrient intake reference values and apply on a global scale to assess intakes across populations (8). The approach incorporates the framework and terminology recommended by reports from the United Nations University, the National Academies of Sciences, Engineering, and Medicine (NASEM), the Institute of Medicine (IOM), and the European Food Safety Authority (EFSA). A recent study, Beal et al. (9) developed global food composition database and calculated recommended nutrient intakes for five populations groups with varying nutritional requirements. In addition, ratings of micronutrients across different food sources were calculated. In brief, the top sources of micronutrients were organ meat, dark leafy vegetables, crustaceans, bivalves (clams, mussels, oysters, and scallops), goat, beef, lamb, fish and milk. Interestingly, foods promoted as nutrient dense, including many fruit and vegetables and whole grains, are not particularly dense in bioavailable micronutrients. These foods provide nutritional benefits beyond specific nutrients, such as fibre, which is important for the gut microbiota.

Integrating nutrients, through better food choices i.e., less ultra-processed food consumption, and foods that have a higher nutrient density is vital for everyone, not just those with CVD. High diet quality messages is an essential component of dietary recommendations, national food policies and health promotion. Specifically, being overfed and/or undernourished is a phenotype, that in our opinion, requires more scientific and clinical discussion and empirical testing in well-designed clinical outcome studies. This is particularly given recent (and worryingly) worldwide high obesity statistics, consumption of nutrient poor processed food and the displacement of traditional eating practices. Nutritional screening in obesity, through diet quality screeners is absolutely key (10) and understanding the barriers, motivators and enablers to improve nutritional status is a priority.
